# Bloodstream Infections: Comparison of Diagnostic Methods and Therapeutic Consequences between a Hospital in a Resource-Limited Setting and Two French Hospitals

**DOI:** 10.3390/microorganisms11092136

**Published:** 2023-08-23

**Authors:** Racha Eid, Jean-Ralph Zahar, Chahrazed Ait Ali, Assaf Mizrahi, Racha Ibrahim, Emeline Banh, Habib Halouani, Françoise Jauréguy, Benoit Pilmis, Rindala Saliba

**Affiliations:** 1 Clinical Microbiology Department, Hotel Dieu de France Teaching Hospital, Saint-Joseph University of Beirut, Beirut 1100, Lebanon; rashaseid@gmail.com (R.E.); rachabrahim@gmail.com (R.I.); rindala.saliba@hdf.usj.edu.lb (R.S.); 2 Clinical Microbiology Department, Avicenne Hospital, 93000 Bobigny, France; chahrazed.aitali@aphp.fr (C.A.A.); habib.halouani@gmail.com (H.H.); francoise.jaureguy@aphp.fr (F.J.); 3 UMR1137-IAME, Inserm, Paris Cite University, 75006 Paris, France; 4 Clinical Microbiology Department, Groupe Hospitalier Paris Saint-Joseph, 75014 Paris, France; amizrahi@ghpsj.fr (A.M.); emeline.banh@universite-paris-scalay.fr (E.B.); bpilmis@ghpsj.fr (B.P.)

**Keywords:** bloodstream infections, resource-limited countries, rapid microbiological methods, antimicrobial resistance, clinical outcomes

## Abstract

In recent years, the diagnosis of bloodstream infections has been complemented by rapid microbiological methods, unattainable to most clinical laboratories in resource-limited settings. We evaluated the impact of their shortage on antibiotic therapy adequacy. We conducted a prospective multicenter cohort study including 150 adult Gram-negative bacilli bacteremia episodes, evenly distributed across three university hospitals: one in Lebanon, a resource-limited setting, and two in France, a resource-rich setting. Previous colonization by multidrug-resistant organisms (MDRO) was significantly more prevalent among the Lebanese than the French group of patients (16/50 vs. 5/100; *p* < 0.01). Bloodstream infections by carbapenemase-producing *Enterobacterales* and other MDRO were higher among the Lebanese than the French group of patients (25/50 vs. 12/100; *p* < 0.01). For the French group, rapid identification of species and mechanisms of resistance significantly shortened turnaround time for definitive laboratory diagnosis and increased antibiotic therapy adequacy. No statistically significant differences were noted in targeted antibiotic therapy between the two groups. This study suggests that, in settings where bacterial resistance is prevalent, rapid microbiological methods have not provided any additional value. The clinical and economic impact of rapid microbiological methods will likely depend on local CPE, VRE, and other MDRO epidemiology and are areas for future research.

## 1. Introduction

Bloodstream infections are severe but treatable conditions requiring prompt microbial identification and susceptibility testing to make the most informed therapeutic decision [1]. In fact, timely reporting of identification and antimicrobial susceptibility testing results allow for the optimization of antimicrobial treatment, leading subsequently to enhanced patient outcomes, reduction in hospital stays, and reduction of exposure to unnecessary broad-spectrum agents [2]. This could consequently reduce the occurrence of infections due to *Clostridioides difficile* and to resistant pathogens [3]. Moreover, the Infectious Diseases Society of America recognizes the need for rapid diagnostics, and sepsis guidelines emphasize the importance of appropriate antimicrobial therapy initiated as soon as possible in preventing deaths [4,5]. In recent years, blood cultures and traditional antimicrobial susceptibility testing have been supplemented by rapid microbiological methods such as nucleic acid hybridization, amplification, and mass spectrometry [6]. Not only do these methods allow for bacterial identification with high sensitivity and specificity for targeted organisms, but they also detect antibiotic resistance and shorten the turnaround time for the laboratory diagnosis of bloodstream infections, compared to conventional methods [7,8]. Direct bacterial identification from blood culture broth using matrix-assisted laser desorption ionization time-of-flight mass spectrometry (MALDI-TOF MS), multiplex polymerase chain reaction assays, or microarray technologies are promising approaches as they reduce time to appropriate antibiotic therapy and hospital costs [9,10]. While these techniques help identify the causative pathogens and detect resistance profiles in sepsis, they do not allow for susceptibility testing to specific antibiotics, nor does the presence of resistance genes always predict resistance [11]. Despite the widespread use of these methods, they are deemed unattainable to most clinical laboratories located in resource-limited settings. For example, Lebanon is a country facing an economic crisis with consequences on the health sector hindering the access of microbiological laboratories to such methods. In addition, this situation has been worsened by a mass exodus of competent and experienced healthcare workers [12]. In this context, the aim of this present study is to assess the impact of a shortage in rapid microbiological methods on antibiotic therapy adequacy. In this present study, rapid microbiological methods encompass direct bacterial identification and rapid detection of resistance profiles from blood culture broth using MALDI-TOF MS and rapid colorimetric methods, respectively. Therefore, we compared antibiotic therapy adequacy in a resource-rich setting to those in a limited-resource one.

## 2. Materials and Methods

We conducted a prospective cohort study, from January to June 2022, in three university hospitals: Hôtel-Dieu de France de Beyrouth, a Lebanese 450-bed hospital; Avicenne Hospital, a French 500-bed hospital; and Saint-Joseph Hospital, a French 600-bed hospital. A total of 150 patients were consecutively enrolled, evenly distributed among the three hospitals. All patients were aged 18 and older, hospitalized, and presented a Gram-negative bacilli bacteremia. Immunocompromised patients and patients with known drug allergies were excluded from this study. Once a blood culture was signaled positive for growth by the automated systems (BACTEC^®^ BD Diagnostics, Franklin Lakes, NJ, USA, in Hôtel-Dieu de France de Beyrouth and in Avicenne Hospital, and BacT/ALERT^®^ VIRTUO^®^ bioMérieux Marcy l’Etoile, France, in Saint-Joseph Hospital), subcultures onto specific media plates and a Gram stain were performed. Gram stain results were immediately communicated to the treating physician and exported into the electronic information system. Bacterial identification was achieved by macroscopic characteristics of colonies accompanied by biochemical tests using API^®^ bioMérieux systems France, in Beirut, Lebanon or by Bruker^®^ MALDI-TOF MS, NJ, USA, applied directly on positive blood broths or on colonies in Paris and Bobigny, France. Rapid colorimetric methods, such as the BetaLACTA^®^ Test (BLT) Bio-Rad^®^ (Hercules, CA, USA), which help determine resistance mechanisms were performed exclusively in France, as described [13]. Antimicrobial susceptibility testing was performed on Mueller Hinton agar by the Kirby-Bauer disk diffusion method after dilution of blood culture broth or by inoculation from a subculture plate, as recommended by CA-SFM/EUCAST. Antimicrobial susceptibility testing was determined using CA-SFM/EUCAST breakpoints. Extended-spectrum beta-lactamase-producing *Enterobacterales* (ESBL-PE) strains were detected by the double-disk synergy test when bacteria were susceptible to cefoxitin (10 µg) [14]. Carbapenemase-producing *Enterobacterales* (CPE) strains were detected when they showed resistance to ertapenem, in accordance with the CA-SFM/EUCAST algorithm, and confirmed using MASTDISCS^®^ Combi Carba plus UK, in Beirut, Lebanon, or Cepheid Xpert Carba-R^®^ Sunnyvale, CA, USA, assay in Paris and Bobigny, France [15,16,17]. We assessed antibiotic therapy adequacy at three different timepoints: empirical antibiotic therapy before blood culture positivity, optimal antibiotic therapy after identification of species and mechanism of resistance, and targeted antibiotic therapy after antibiotic susceptibility testing completion. Antibiotic therapy adequacy was classified as ineffective, adequate, or overused. Ineffective antibiotic therapy occurs when the chosen antibiotic lacks the ability to target the causative bacteria, as it does not align with its sensitivity. Adequate antibiotic therapy occurs when the chosen antibiotic sufficiently targets the susceptibility of the causative bacteria. Overused antibiotic therapy occurs when the chosen antibiotic possesses a broader spectrum of activity than what is needed to target the causative bacteria. For Beta-lactam antibiotic therapy, assessment was performed according to Weiss et al. [18]. For non-Beta-lactam antibiotic therapy, we considered the following as adequate: Aminoglycosides, Levofloxacin, and Ciprofloxacin on *Pseudomonas* spp., or Fluoroquinolones on *Enterobacterales* in the absence of an alternative. In the absence of the availability in Lebanon of molecules such as aztreonam, ceftazidime-avibactam, and cefiderocol, colistin was considered the best available therapy for the management of these infections. We have decided to classify the use of colistin monotherapy for the treatment of CPE-related infections as “undetermined”. As for CPE infections resistant to ceftazidime-avibactam and fluoroquinolones, we considered the combination of colistin and aminoglycoside as adequate as they were the only available and microbiologically active agents. We also evaluated antibiotic therapy adequacy according to clinical judgement which takes factors such as the causative agent, route of infection, bacterial epidemiology, type of infection, and colonization into consideration.

Demographic data such as age, gender, and ward of hospitalization were collected. Clinically, various data were collected such as route of infection, type of infection (community-acquired: infection acquired outside a healthcare setting; or healthcare-associated: infection contracted in a healthcare setting or associated with medical devices or occurring at surgical sites), and patient-related factors for antibiotic resistance (hospitalization in the last 6 months, antibiotics use in the last 3 months, colonization in the last 12 months with vancomycin-resistant *Enterococcus* (VRE), CPE, and/or other multidrug-resistant organisms (MDRO) such as extended-spectrum beta-lactamase-producing *Enterobacterales* and multidrug-resistant *Pseudomonas aeruginosa*). Microbiological data such as the causative pathogen, mechanism of resistance, time elapsed to genus and species identification, rapid colorimetric assays for mechanisms of resistance, and definitive antibiotic susceptibility testing were collected. Empirical antibiotic therapy as well as eventual adjustments were noted in order to assess antibiotic therapy adequacy at the three different timepoints previously mentioned. In addition, we recorded patients’ outcome parameters, including eventual complications, length of hospital stays, and 30-day mortality. Eventual complications include tissue damage, organ failure, metabolic acidosis, severe hypoxemia, and septic thrombosis.

## 3. Results

### 3.1. Characteristics of Study Population

One hundred and fifty patients were analyzed. Table 1 compares the demographic and clinical data of patients from the French hospitals to patients from the Lebanese hospital. Sex, age, ward of hospitalization, route of bloodstream infection, and type of infection were comparable between the two groups, indicating absence of selection bias in this population-based cohort study. Hospitalization during the past 6 months, administration of antibiotics during the past three months, and previous known colonization by VRE and/or CPE were comparable between the two groups. Known previous colonization by other MDRO was, however, significantly more frequent among patients from Lebanon than among patients from France (32% vs. 5%; *p* < 0.01).

### 3.2. Bacteriological Data

Distribution of the causative agents and their mechanisms of resistance are summarized in Table 2. ESBL-PE isolates were more prevalent in the Lebanese group than in the French group (38% vs. 11%; *p* < 0.01). Six cases of CPE were detected which were all from Lebanon. On the first day of blood culture positivity, identification of the causative pathogens and their respective mechanisms of resistance were unachievable in the Lebanese group, as opposed to the French group. Turnaround time to definitive laboratory diagnosis was significantly faster in the French group.

The incidence of bloodstream infections by CPE and other MDRO was higher among the Lebanese group than among the French group of patients (67.5% and 32.5%; *p* < 0.01). Unlike prior hospitalization, colonization with MDRO in the last 12 months was significantly linked with MDRO bloodstream infections. Empirical antibiotic therapy was significantly more adequate for CPE and other MDRO than non-MDRO. However, no statistically significant differences were found in the adequacy of targeted antibiotic therapy and 30-day mortality (Table 3).

### 3.3. Antibiotic Therapy Adequacy

More adequate empirical antibiotic therapy was observed in the Lebanese group than in the French group (28% vs. 14%; *p* = 0.04). However, based on clinical judgments, no statistically significant differences were noted between the two groups. Concerning optimal antibiotic therapy after identification of species and mechanisms of resistance in France, ineffective antibiotic therapy decreased, while adequate antibiotic usage increased. Antibiotic usage remained unchanged for the Lebanese, where rapid colorimetric or molecular tests were not available. Concerning targeted antibiotic therapy based on antibiotic susceptibility testing, the use of adequate antibiotics was statistically more significant in the Lebanese group. Moreover, the use of unnecessary broad-spectrum antibiotics was statistically more significant in the French group. Otherwise, based on clinical judgments, no statistically significant differences were noted between the two groups in targeted antibiotic therapy. Adequacy of antibiotic therapy remained undetermined for patients who underwent hospital transfers (Table 4).

### 3.4. Patients’ Clinical Outcomes

No statistically significant differences were noted between the two groups relating to eventual complications within a 3-month follow-up, length of hospital stay, and 30-day mortality (Table 5).

A multivariate mortality analysis was performed (Table 6). Infections uncategorized as either community-acquired or healthcare-associated demonstrated a notably higher mortality rate (*p* = 0.01). Furthermore, a statistically significant association was found between mortality rate and the use of antibiotics in the last three months, as well as infections related to CPE (*p* = 0.01 and 0.04, respectively). Patients who received initial effective antibiotic therapy exhibited a notable improved survival rate (*p* = 0.049).

## 4. Discussion

We evaluated the effect of the lack of rapid microbiological methods including direct bacterial identification and rapid detection of resistance profiles from blood culture broth using MALDI-TOF MS and rapid colorimetric methods, respectively. In the absence of these diagnostic methods, identification of the causative pathogen and mechanism of resistance were unachievable at the first day of positivity. While these rapid methods certainly enhance and facilitate the effective and targeted treatment such as the observed increase from 14% to 32% in adequacy following their use in the French group, their lack was not associated with less antibiotic therapy adequacy in our study and had no effect on patients’ clinical outcomes. In fact, we observed more statistically significant bloodstream infections with ESBL-PE and CPE in the Lebanese group, which could explain the adequacy of broad-spectrum antibiotic use in the absence of a rapid detection of resistance. The lack of certain antibiotics from the Lebanese market led to the use of relatively broader-spectrum agents that otherwise would have been reserved for resistant pathogens. For instance, due to the unavailability of piperacillin, a combination of piperacillin-tazobactam was used for the management of piperacillin-susceptible pseudomonas bloodstream infection. In the Lebanese hospital, the lack of proper stewardship implementation, particularly in the absence of proper guidance by infectious disease specialists, may be associated with the use of unnecessary broad-spectrum antibiotics. For example, in our study, a combination of colistin and meropenem was used to treat an ESBL infection susceptible to piperacillin-tazobactam.

Even though comprehensive data remain scarce, previous studies suggest that low-to-middle-income countries (LMICs) including Lebanon are likely to be most affected by the declining effectiveness of antibiotics. LMICs’ higher exposure is by dint of pre-existing developmental and economical challenges and likely deficiencies in the healthcare system [19]. In fact, certain countries in the Mediterranean region and Arabian Peninsula report a high prevalence of CPE [20]. Talaat et al. assessed the burden of antimicrobial resistance in seven countries in the World Health Organization Eastern Mediterranean Region during 2017–2019. In that aim, data on bloodstream infections reported to the Global Antimicrobial Resistance Surveillance System were analyzed. The median proportion of bloodstream infections was highest for carbapenem-resistant *Acinetobacter* spp. (70.3%), compared to 33.9% in the United States and 33.9% in European Union countries, followed by third-generation cephalosporin–resistant *Enterobacterales*, methicillin-resistant *Staphylococcus aureus*, and CPE [21]. High prevalence of antimicrobial resistance in the studied region may be attributed to self-medication by antimicrobial drugs that are readily available over the counter, poor infection prevention and control in healthcare facilities, limited capacities of microbiology laboratories, limited antimicrobial stewardship programs, and geopolitical tensions causing population displacement.

Despite a shortage in rapid diagnostic methods, adequacy was not affected in our study. Although bloodstream infections to resistant pathogens were more prevalent in the Lebanese cohort of patients, empirical therapy was more appropriate. Meanwhile, in the French cohort of patients, targeted antibiotic therapy was overused. Hence, our study showed that, in settings where the prevalence of bacterial resistance is high, rapid microbiological methods have not provided any additional value. Therefore, epidemiological surveillance is of utmost importance since it influences the choice of empirical antibiotic therapy and provides the necessary input for developing therapy guidelines, antimicrobial stewardship programs, and public health interventions. In fact, the choice of empirical antibiotic therapy can be influenced by epidemiologic risk factors, such as a high prevalence of VRE, CPE, or other MDRO, that can influence the probability of antibiotic resistance and likelihood of successful treatment. Mehl et al. prospectively recorded 1995 episodes of bloodstream infections between 2002 and 2013 in a medium-sized Norwegian hospital where antimicrobial resistance was relatively low. In this cohort, appropriate empirical antibiotic therapy was achieved by replacing second- and third-generation cephalosporins with penicillin and gentamicin [22]. In contrast, Pradipta et al. conducted a retrospective observational study on 76 episodes of bloodstream infections during January-December 2011 in an Indonesian hospital, where antimicrobial resistance was considered high. Based on the results of the microbial cultures, antibiotic susceptibility tests and patterns of antibiotic use, 61.35% of the empirical antibiotics used, including third generation cephalosporins, showed resistance rates of more than 50%. This finding should influence the use of appropriate broad-spectrum empirical antibiotic therapy for reducing mortality and morbidity in sepsis patients [23].

On the other hand, the choice of empirical antibiotic therapy may be determined by assessing clinical scoring systems that predict the likely source of infection. Goodman et al. evaluated a clinical decision algorithm predicting ESBL-PE bloodstream infections in 1288 patients at Johns Hopkins Hospital. Of the 194 patients with ESBL-PE bloodstream infections, 68 (35%) received empirical carbapenem therapy within 6 h after causative pathogen identification. This tool correctly identified one-third of the original 68 patients, as well as an additional 78 cases. Use of the clinical decision algorithm would have increased by 50% ESBL-PE detection during the empirical treatment, justifying the use of agents covering ESBL-PE [24].

The role of rapid diagnostics in bloodstream infections has been subject to a number of publications. The RAPIDO multicenter randomized control trial, including 8628 patients across seven National Health Service acute hospital trusts in England and Wales, compared the impact of rapid microbial identification and conventional methods on clinical outcomes in bloodstream infection. In this study, conventional methods included performing a Gram stain and microscopy followed by culture to isolate individual species, and biochemical tests to identity and reculture to test antimicrobial susceptibility. In the rapid diagnosis arm, direct MALDI-TOF MS after extraction was used in addition to conventional methods. Findings concerning 30-day mortality and length of hospital stay are in concordance with ours, despite several limitations in our study. No significant differences were observed in total antimicrobial consumption, *Clostridioides difficile* infection, time to resolution of fever, time to discharge from hospital, or de-escalation of broad-spectrum therapy between the two groups. While it was shown that microbial identification was quicker in the rapid diagnosis group, time to effective antimicrobial therapy was no shorter [25]. Findings concerning 30-day mortality and length of hospital stay are in concordance with ours. In contrast, a meta-analysis of 31 trials assessed the impact of a rapid diagnostic test compared with conventional microbiology methods in improving clinical outcomes in patients with bloodstream infections. It found a significant mortality benefit with rapid diagnostic tests vs. conventional methods when these were coupled with an antimicrobial stewardship program (OR, 0.64; 95% CI: 0.51–0.79). However, this advantage was lost in the absence of antimicrobial stewardship supporting infrastructure. This may be attributed to the real-time notification and intervention of antimicrobial stewardship teams which consequently improve time to effective therapy compared to reporting of microbiology results alone [26]. Indeed, recent data from LMICs have suggested that implementing rapid diagnostic tests in the absence of an antimicrobial stewardship program results in limited benefits with regard to antimicrobial use or clinical outcomes [27,28]. A 2021 Cochrane Review sought to evaluate the effect of rapid genotypic and phenotypic susceptibility testing versus standard microbial susceptibility testing on treatment of bloodstream infections. It was shown that rapid susceptibility tests that identify appropriate antibiotics quickly may make little to no difference in 30-day mortality, in length of hospital stay, and in time to appropriate antibiotic therapy, but the certainty of evidence is low. While it remained unclear whether phenotypic rapid susceptibility tests reduce time to appropriate antibiotic therapy, genotypic rapid susceptibility tests made little or no difference. Moreover, rapid identification along with rapid susceptibility testing may make little or no differences in time to appropriate antibiotic therapy [29].

On another note, despite the use of rapid microbiological methods, the overuse of broad-spectrum antibiotics increased from 40% to 49% in the French hospitals. Also, the overuse of broad-spectrum antibiotics in targeted antibiotic therapy, based on clinical judgments, was observed in 19% and 28% of the included patients in the French and Lebanese hospitals, respectively (Table 4). Inappropriate use of broad-spectrum antibiotics when they are not needed has repercussions on the individual level and causes long-term collective risks, with antimicrobial resistance as an outcome. At the individual level, the use of such antibiotics leads to dysbiosis, which offers resistant bacteria the selective advantage to proliferate and increases susceptibility to *Clostridioides difficile* infections [30]. However, this is subject to individual factors such as initial microbiota composition, dietary habits, and physiological modifications related to the patient’s clinical situation [31,32]. At a collective level, in a hospital setting or the community, antibiotic-resistant bacteria would spread to other persons causing a real burden [33].

Our multicenter study has some limitations. Its major limitation is the small number of patients included, particularly in the resource-limited hospital, which may explain the observed absence of difference between the two studied groups. Therefore, our findings should be investigated on a larger sample of patients. Also, this study was conducted in only one university hospital in Lebanon. Even though Hôtel-Dieu de France is a major hospital in Beirut, the findings of our study may not be generalized to other hospitals, especially in the absence of antimicrobial stewardship programs and national recommendations for bloodstream infections. Moreover, differences in laboratory workflow and infectious disease consultations between the French and Lebanese hospitals were not taken into account. Finally, the absence of significant differences in 30-day mortality between the two groups is difficult to defend insofar as we have not taken into account the multiple confounding factors that may be involved.

In conclusion, bloodstream infections are life-threatening situations where rapid pathogen identification and antibiotic susceptibility testing enable clinicians to optimize antibiotic therapy, consequently leading to better patient clinical outcomes. Advancements in rapid diagnostics shorten laboratory turnaround time. Co-implementation of rapid microbiological methods and antimicrobial stewardship interventions may significantly enhance patients’ outcomes. In our experience and in situations of a high prevalence of resistance, rapid microbiological methods have not provided any additional value. The clinical and economic impact of rapid microbiological methods will likely depend on local CPE, VRE, and other MDRO epidemiology and are areas for future research.

## Figures and Tables

**Table 1:** Demographic and clinical data.

Characteristics	France	Lebanon	*p*-Value
Total	100	50	
Males, n (%)	59 (59)	27 (54)	0.6
Age, median [IQR]	71 [56–81]	72 [62–82]	0.8
Ward of hospitalization, n (%)			
Medical	79 (79)	41 (82)	0.82
Intensive care unit	15 (15)	7 (14)	1
Surgery	6 (6)	2 (4)	0.71
Route of infection, n (%)			
Urinary	37 (37)	22 (44)	0.47
Digestive	24 (24)	15 (30)	0.43
Pulmonary	15 (15)	4 (8)	0.3
Catheter	11 (11)	6(12)	1
Surgical site	2 (2)	3 (6)	0.33
Primary bacteremia	6 (6)	0 (0)	1
Cutaneous	3 (3)	0 (0)	0.55
Endocarditis	1 (1)	0 (0)	1
Skeletal	1 (1)	0 (0)	1
Type of infection, n (%)			
Community-acquired	52 (52)	28 (56)	0.72
Healthcare-associated	42 (42)	20 (40)	0.86
Unknown	6 (6)	2 (4)	0.71
Hospitalization in the last 6 months, n (%)	49 (49)	30 (60)	0.22
Antibiotics use in the last 3 months, n (%)	25 (25)	20 (40)	0.08
Colonization with MDRO in the last 12 months, n (%)	5 (5)	16 (32)	<0.01
Colonization with VRE and/or CPE in the last 12 months, n (%)	3 (3)	5 (10)	0.11

CPE, carbapenemase-producing *Enterobacterales*; IQR, interquartile range; MDRO, multidrug-resistant organisms; VRE, vancomycin-resistant *Enterobacterales.*

**Table 2:** Bacteriological data.

Characteristics	France	Lebanon	*p*-Value
Causative pathogen			
*Escherichia coli*, n (%)	50 (50)	31 (62)	0.22
*Klebsiella pneumoniae*, n (%)	12 (12)	9 (18)	0.32
*Enterobacter* spp., n (%)	10 (10)	0 (0)	0.03
*Pseudomonas aeruginosa*, n (%)	13 (13)	4 (8)	0.42
Other, n (%)	15 (15)	6 (12)	0.8
Mechanism of resistance			
ESBL, n (%)	12 (12)	19 (38)	<0.01
AmpC, n (%)	4 (4)	3 (6)	1
CPE, n (%)	0 (0)	6 (12)	0.002
First day of positivity			
Time to positivity (hours), median [IQR]	10.6 [6–11.65]	12 [9.35–17.8]	<0.001
Gram stain	100 (100)	43 (86)	0.0003
Mobility	36 (36)	43 (86)	0.0001
Genus identification	81 (81)	0 (0)	0.0001
Species identification	74 (74)	0 (0)	0.0001
BetaLACTA^®^ test	78 (78)	0 (0)	0.0001
Positive rapid test	11 (11)	0 (0)	0.01
Identification of mechanism of resistance	70 (70)	0 (0)	0.01
AST by disk diffusion	100 (100)	33 (66)	0.0001
AST reading	25 (25)	0 (0)	0.0001
Second day of positivity			
Genus identification	100 (100)	28 (56)	0.0001
Species identification	99 (99)	25 (50)	0.0001
BetaLACTA^®^ test	3 (3)	0 (0)	0.24
AST performed	36 (36)	15 (30)	0.58
Time to genus identification (h), median [IQR]	26.2 [13.8–32.4]	36.5 [24.3–48.2]	<0.001
Time to species identification (h), median [IQR]	26.4 [14.4–32.2]	42.6 [24.5–48.5]	<0.001
Time to mechanism of resistance identification (h), median [IQR]	29 [15.3–33]	37.5 [23.3–49.2]	0.0004
Time to definitive laboratory result (h), median [IQR]	41.6 [33–55]	43.6 [24.9–50]	0.008

AmpC, AmpC beta-lactamase; AST, antimicrobial susceptibility testing; CPE, carbapenemase-producing *Enterobacterales*; ESBL, extended-spectrum beta-lactamase; IQR, interquartile range.

**Table 3:** Characteristics of CPE and other MDRO vs. non-MDRO bloodstream infections.

Characteristics	CPE, Other MDRO	Non-MDRO	*p*-Value
Total	37	113	
Males, n (%)	20 (54)	66 (58.4)	0.83
Location, n (%)			
France	12 (32.5)	88 (77.9)	<0.01
Lebanon	25 (67.5)	25 (22.1)	
Ward of hospitalization, n (%)			
Medical	28 (75.7)	92 (81.4)	0.48
Surgery	3 (8.1)	5 (4.4)	0.4
Intensive care	6 (16.2)	16 (14.2)	0.79
Route of infection, n (%)			
Urinary	18 (48.6)	41 (36.3)	0.24
Digestive	11 (29.7)	28 (24.8)	0.66
Catheter	4 (10.8)	13 (11.5)	1
Pulmonary	2 (5.4)	17 (15)	0.16
Surgical site	2 (5.4)	3 (2.7)	0.59
Primary bacteremia	0 (0)	6 (5.4)	0.33
Cutaneous	0 (0)	3 (0)	1
Endocarditis	0 (0)	1 (0)	1
Skeletal	0 (0)	1 (0)	1
Type of infection, n (%)			
Community-acquired	17 (46)	63 (55.7)	0.34
Healthcare-associated	18 (48.6)	44 (38.9)	0.33
Unknown	2 (5.4)	6 (5.4)	1
Hospitalization in the last 6 months, n (%)	20 (54)	59 (52.2)	1
Antibiotics use in the last 3 months, n (%)	17 (46)	28 (24.7)	0.02
Colonization with VRE and/or CPE in the last 12 months, n (%)	4 (10.8)	4 (3.5)	0.1
Colonization with other MDRO in the last 12 months, n (%)	14 (37.8)	7 (6.2)	<0.01
Species			
*Escherichia coli*, n (%)	28 (75.7)	53 (46.9)	0.002
*Klebsiella pneumoniae*, n (%)	7 (18.9)	14 (12.4)	0.41
*Enterobacter* spp., n (%)	1 (2.7)	9 (8)	0.45
*Pseudomonas aeruginosa*, n (%)	0 (0)	17 (15)	0.007
Other, n (%)	1 (2.7)	20 (17.7)	0.02
Empirical adequate antibiotic therapy, n (%)	20 (54)	31 (27.4)	0.004
Targeted adequate antibiotic therapy, n (%)	2 (5.4)	5 (4.4)	1
30-day mortality, n (%)	6 (16.2)	16 (14.1)	0.79

CPE, carbapenemase-producing *Enterobacterales*; MDRO, multidrug-resistant organisms such as Extended-spectrum beta-lactamase-producing *Enterobacterales* and multidrug-resistant *Pseudomonas aeruginosa*.

**Table 4:** Adequacy of antibiotic therapy.

Characteristics	France	Lebanon	*p*-Value
Empirical antibiotic therapy, n (%)			
- Antibiotic susceptibility testing			
Ineffective	41 (41)	14 (28)	0.15
Adequate	14 (14)	14 (28)	0.04
Overused	40 (40)	21 (42)	0.86
Undetermined	5 (5)	1 (2)	0.66
- Clinical judgment-based			
Ineffective	38 (38)	13 (26)	0.2
Adequate	48 (48)	26 (52)	0.72
Overused	12 (12)	9 (18)	0.36
Undetermined	2 (2)	1 (2)	1
Optimal antibiotic therapy, n (%)			
- Antibiotic susceptibility testing			
Ineffective	16 (16)	14 (28)	0.08
Adequate	32 (32)	14 (28)	0.7
Overused	49 (49)	21 (42)	0.48
Undetermined	3 (3)	1 (2)	0.1
Targeted antibiotic therapy, n (%)			
- Antibiotic susceptibility testing			
Ineffective	8 (8)	1 (2)	0.27
Adequate	34 (34)	29 (58)	0.008
Overused	52 (52)	17 (34)	0.03
Undetermined	6 (6)	3 (6)	1
- Clinical judgment-based			
Ineffective	9 (9)	1 (2)	0.42
Adequate	68 (68)	34 (68)	1
Overused	19 (19)	14 (28)	1
Undetermined	2 (2)	1 (2)	1

**Table 5:** Clinical outcomes.

Characteristics	France	Lebanon	*p*-Value
Complications within 3 months, n (%)	3 (3)	0 (0)	0.55
Length of hospital stay (days), median [IQR]	11 [6–17.5]	11 [8–18]	0.97
30-day mortality, n (%)	13 (13)	9 (18)	0.46
IQR, interquartile range			

**Table 6:** Multivariate mortality analysis.

Characteristics	Death	Survival	*p*-Value
Total	22	128	
Males, n (%)	11 (50)	77 (60.1)	0.48
Location, n (%)			
France	13 (59.1)	87 (68)	0.46
Lebanon	9 (40.9)	41 (32)	
Ward of hospitalization, n (%)			
Medical	17 (77.3)	103 (80.4)	0.77
Surgery	0 (0)	8 (6.3)	0.60
Intensive care	5 (22.7)	17 (13.3)	0.32
Route of infection, n (%)			
Urinary	5 (22.7)	54 (42.2)	0.1
Digestive	5 (22.7)	34 (26.6)	0.79
Catheter	5 (22.7)	12 (9.4)	0.13
Pulmonary	5 (22.7)	14 (10.9)	0.15
Surgical site	0 (0)	5 (3.9)	1
Primary bacteremia	2 (9.2)	4 (3.1)	0.21
Cutaneous	0 (0)	3 (2.3)	1
Endocarditis	0 (0)	1 (0.8)	1
Skeletal	0 (0)	1 (0.8)	1
Type of infection, n (%)			
Community-acquired	8 (36.4)	72 (56.3)	0.1
Healthcare-associated	10 (45.5)	52 (40.6)	0.81
Unknown	4 (18.1)	4 (3.1)	0.01
Hospitalization in the last 6 months, n (%)	14 (63.6)	65 (50.8)	0.35
Antibiotics use in the last 3 months, n (%)	12 (54.5)	33 (25.7)	0.01
Colonization with VRE and/or CPE in the last 12 months, n (%)	2 (9.2)	6 (4.7)	0.33
Colonization with other MDRO in the last 12 months, n (%)	2 (9.2)	19 (14.8)	0.74
Species			
*Escherichia coli*, n (%)	10 (45.4)	71 (55.5)	0.48
*Klebsiella pneumoniae*, n (%)	5 (22.7)	16 (12.5)	0.19
*Enterobacter* spp., n (%)	1 (4.5)	9 (7)	1
*Pseudomonas aeruginosa*, n (%)	3 (13.7)	14 (10.9)	0.71
Other, n (%)	3 (13.7)	18 (14.1)	1
MDRO	8 (36.3)	29 (22.6)	0.18
ESBL, n (%)	5 (22.7)	26 (20.3)	0.77
CPE, n (%)	3 (13.6)	3 (2.3)	0.04
Initial effective antibiotic therapy, n (%)	13 (59)	103 (80.4)	0.049
Final effective antibiotic therapy, n (%)	19 (86.3)	113 (88.2)	0.73

CPE, carbapenemase-producing *Enterobacterales*; MDRO, multidrug-resistant organisms such as Extended-spectrum beta-lactamase-producing *Enterobacterales* and multidrug-resistant *Pseudomonas aeruginosa*.

## Data Availability

Not applicable.
